# Case Report: A Case of Wood-Smoke–Related Pulmonary Disease

**DOI:** 10.1289/ehp.8489

**Published:** 2006-01-24

**Authors:** Janet V. Diaz, Jonathan Koff, Michael B. Gotway, Stephen Nishimura, John R. Balmes

**Affiliations:** 1 Department of Medicine, Division of Pulmonary and Critical Care Medicine,; 2 Department of Radiology and; 3 Department of Pathology, San Francisco General Hospital, University of California at San Francisco, San Francisco, California, USA

**Keywords:** biomass combustion, domestically acquired particulate lung disease, hut lung, indoor air pollution, wood smoke

## Abstract

**Context:**

Biomass serves as a major fuel source for > 50% of the world’s population. The global burden of disease attributed to indoor air pollution from biomass combustion accounts for approximately 3% of worldwide disability-adjusted life-years lost. This is due to pneumonia in children and chronic obstructive pulmonary disease and lung cancer in women.

**Case Presentation:**

A 53-year-old man from Mexico was referred to the pulmonary clinic for evaluation of chronic productive cough and pulmonary nodules. In his youth, he worked at a charcoal plant in Mexico, where he burned wood and was exposed to massive amounts of smoke. His evaluation revealed thickened bronchovascular bundles with nodules on thoracic computed tomography, dark black plaques in large airways on bronchoscopy, and carbon-laden macrophages and fibrotic scars on lung biopsy.

**Discussion:**

The patient was diagnosed with “hut lung,” a term that refers to the noninfectious, nonmalignant respiratory manifestations of chronic, high-level exposures to biomass smoke. This is the first reported case of hut lung associated with charcoal production. This case highlights that histopathologic abnormalities of the lung parenchyma may be present in patients with only mild symptoms and that clinical progression is likely a function of both the duration and intensity of exposure.

**Relevance to Clinical Practice:**

As residents of lesser developed countries continue to be exposed to high levels of biomass smoke at work or at home and continue to immigrate to developed countries, it is important that health care providers in developed countries be aware of biomass-smoke–related pulmonary disease.

## Case Presentation

**Figure 1:**
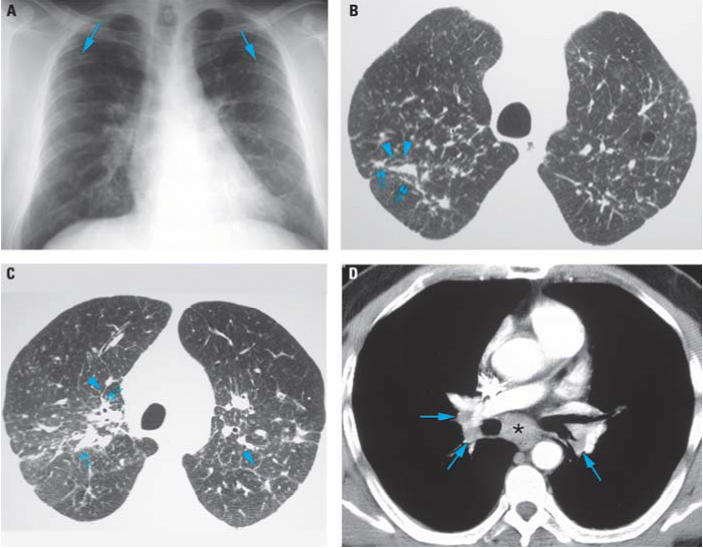
Chest radiograph (*A*) and HRCT images (*B–D*) of patient’s lungs. (*A*) Frontal chest radiograph showing mild symmetric linear and reticular opacities (arrows) in the upper lobes bilaterally; these opacities are associated with bilateral hilar prominence, suggesting lymphadenopathy. Note the upper lobe distribution of the findings as well as the absence of associated pleural thickening. (*B*) Axial HRCT image through the lung apices shows bilateral, patchy bronchovascular thickening with a nodular appearance (small double arrows). Nodular interlobular septal thickening is also present (arrowheads). (*C*) Axial HRCT image through the upper lungs slightly caudal to (*B*) shows bilateral, patchy bronchovascular thickening with a nodular appearance (small double arrows). Mild interlobular septal thickening is again present (arrowheads). Note the posterior distribution of abnormalities. *D*) Contrast-enhanced axial CT image shows subcarinal (*) and mild bilateral hilar (arrows) lymphadenopathy.

A 53-year-old man from Mexico was referred to the San Francisco General Hospital chest clinic for the evaluation of a chronic cough and pulmonary nodules. His respiratory symptoms began in 1985 as an intermittent cough. The cough gradually progressed over the years and was now chronic, productive of yellow sputum, and associated with mild dyspnea. In 1997, he was diagnosed with asthma in Mexico and treated with inhaled bronchodilators with minimal relief. In 2004, he immigrated to the United States and was seen by a primary care provider for these symptoms and treated with a steroid inhaler without relief. At that evaluation, a chest radiograph was performed (Figure 1A), and he was then referred to a chest clinic.

At his chest clinic visit, he denied any current fevers, chills, sweats, weight loss, eye pain, arthralgias, rash, or sinusitis. He had no pets or mold at home, no recent travel, and no environmental tobacco smoke exposures. He reported no other medical conditions. He denied any tobacco, alcohol, or illicit drug use.

His occupational history was significant for work at a charcoal production plant in Mexico when he was 18–26 years of age. He spent 10–12 hr/day burning wood to make charcoal, and he stood very near the fire. He described the environment as being very smoky. He did not use any protective respiratory masks. He denied mining, construction, or asbestos-related occupations. For the past year, he had been working in a restaurant as a dishwasher.

On physical examination, he appeared healthy without any signs of distress and had normal vital signs and an oxygen saturation of 97%. The remainder of the examination was normal except for bilateral forced expiratory wheezes. He had no signs of pulmonary hypertension, right heart failure, or clubbing. Routine laboratory tests revealed a complete blood count and blood chemistry panel that were normal. Three sputum samples for mycobacteria had no growth at 56 days. Serologies for fungal diseases were negative.

We performed thoracic high-resolution computed axial tomography (HRCT), using 7 mm helical technique after intravenous contrast administration followed by high-resolution imaging, using 1 mm collimation every 1 cm from lung apex to base (Figure 1B–D). Pulmonary function test results were as follows: forced vital capacity (FVC), 3.46 L (91%); forced expiratory volume in 1 sec (FEV_1_), 2.31 L (74%); FEV_1_:FVC ratio, 67; total lung capacity, 5.27 L (95%); residual volume, 1.57 L (91%); diffusion capacity for carbon monoxide, 23.1 mL/mmHg/min (81%); and diffusion capacity for CO corrected for total lung capacity by single breath, 4.61 mL/mmHg/L (87%). Arterial blood gases were normal.

**Figure 2:**
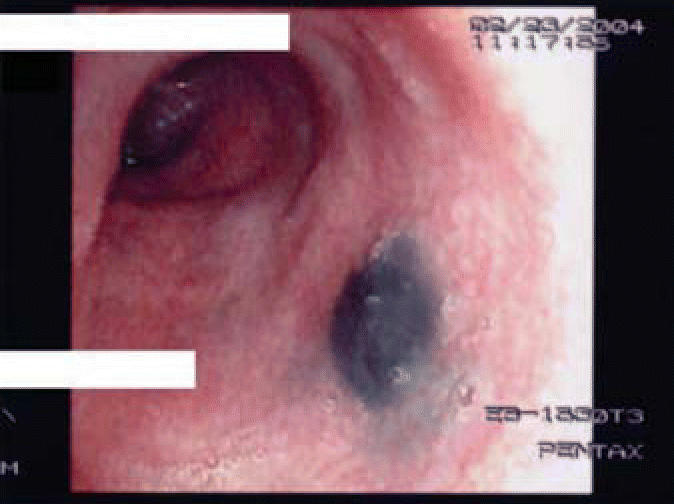
Bronchoscopy demonstrated a black and gray plaque in the left mainstem bronchus. White bars cover the patient’s name and medical record number.

**Figure 3:**
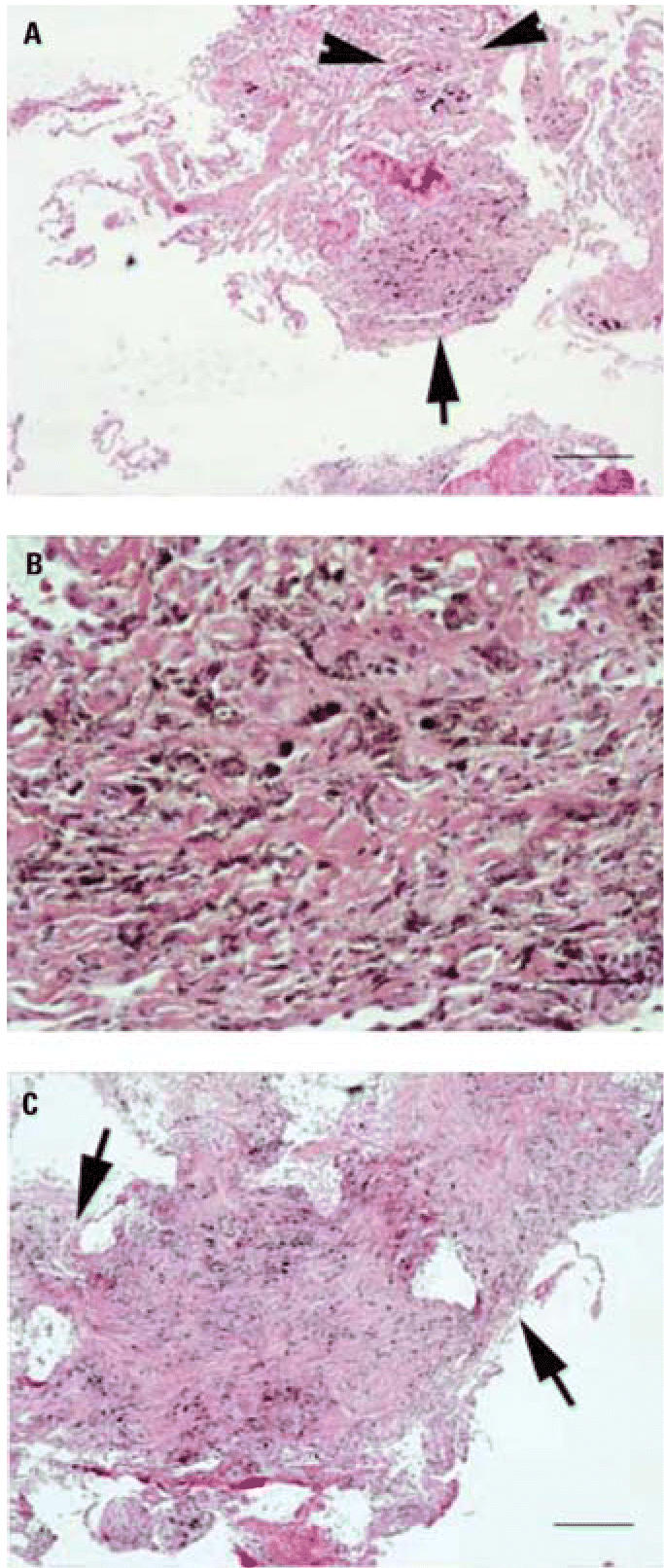
Histopathologic features of transbronchial biopsies. (*A*) Anthracotic pigment accumulating along alveolar septae (arrowheads) and within a pigmented dust macule (single arrow); bar = 200 μm. (*B*) A high-power photomicrograph containing a mixture of fibroblasts and carbon-laden macrophages; bar = 50 μm. (*C*) A fibrotic scar is seen projecting into surrounding interstitial septae giving a stellate appearance (arrowheads); bar = 100 μm.

The patient underwent bronchoscopy for the suspected diagnosis of sarcoidosis. Bronchoscopy demonstrated a large black and gray plaque in the left mainstem bronchus (Figure 2) and multiple smaller gray and black deposits in the left upper lobe, left lower lobe, and right lower lobe. Transbronchial biopsies were obtained (Figure 3). Bronchoalveolar lavage fluid showed normal cell differential counts.

The patient was diagnosed with “hut lung,” or domestically acquired particulate lung disease (Gold et al. 2000; Grobbelaar and Bateman 1991).

## Discussion

The term “hut lung” has been used to describe a wide spectrum of clinical manifestations including chronic bronchitis (CB), chronic obstructive pulmonary disease (COPD), and interstitial lung disease associated with high level exposures to biomass smoke. This is the first reported case of hut lung associated with charcoal production. It highlights the characteristic findings of hut lung that have been reported in previous case series of women from the developing world who cook with biomass indoors (Gold et al. 2000; Grobbelaar and Bateman 1991; Ozbay et al. 2001; Sandoval et al. 1993). This case clearly demonstrates that physiologic, radiographic, and histopathologic abnormalities may persist years after removal from exposure. Hut lung is likely underdiagnosed because those at risk have poor access to health care. This raises the importance of recognizing risk for this disease among biomass-smoke–exposed populations.

Biomass is any material derived from living or recently living material, including animal dung, twigs, grass, crop wastes, wood, and charcoal. More than half of the world’s population uses biomass as a major source of energy for cooking, baking, and heating. This occurs predominantly in rural areas of lesser developed countries where biomass is burned indoors. Because homes are poorly ventilated and this fuel source is inefficient, requiring fires to be kept going for many hours a day, women and their infant children are exposed to years of daily smoke (Bruce et al. 2000; Ezzati and Kammen 2002; Manuel 2003).

Biomass combustion releases smoke that contains particulate matter (PM), CO, nitrogen oxides, formaldehyde, and polyaromatic hydrocarbons (Boman et al. 2003; Zelikoff et al. 2002). Indoor biomass combustion creates massive amounts of indoor air pollution. Measurements of 24-hr mean indoor levels of PM_10_ (particles with mass median aerodynamic diameter of < 10 μm) have been reported between 300 and 30,000 μg/m^3^ and CO between 2 and 500 ppm: these levels are 2–200 times higher than the U.S. Environmental Protection Agency regulations for outdoor air pollutants (Bruce et al. 2000; Ezzati and Kammen 2002; Manuel 2003). PM_10_ can bypass the filtering system of the nasal and oral cavity to either deposit on the mucosa of large- and medium-sized airways (coarse PM) or deposit deep in the alveoli (fine PM), and thus is able to affect respiratory health (Balmes and Tager 2000). Exposure to high levels of outdoor PM_10_ is independently related to lung cancer and cardiopulmonary mortality [World Health Organization (WHO) 2002].

There are limited data on the mechanisms by which biomass smoke causes chronic pulmonary disease. Both macrophage dysfunction and increased activity of matrix metalloproteinase (MMP) have been reported. Rabbits exposed to acute wood smoke had impaired macrophage phagocytic function, surface adherence (Fick et al. 1984), and reduced bacterial clearance (Zelikoff et al. 2002). Rats exposed to chronic wood smoke developed mild bronchiolitis with epithelial cell hyperplasia and hypertrophy, alveolar septal thickening, and mild emphysema (Lal et al. 1993). Bronchoalveolar lavage samples from human subjects with COPD associated with wood-smoke exposure demonstrated significantly higher MMP activity, specifically pro-MMP-2, pro-MMP-9, and MMP-9, and gene expression of MMP-2 and MMP-12, when compared with healthy controls (Montano et al. 2004). Controlled animal and human exposures to concentrated ambient particulates have demonstrated induction of pulmonary inflammation (Saldiva et al. 2002).

There is strong epidemiologic evidence that biomass smoke is associated with the development of CB and COPD. The prevalence rates of CB in communities exposed to indoor biomass smoke have been reported to be high (Albalak et al. 1999; Behera and Jindal 1991; Golshan et al. 2002; Pandey 1984; Pandey et al. 1985; Perez-Padilla et al. 1996). In rural Nepal, the prevalence rate of CB was 19.8% in nonsmoking women who spent more than 4 hr/day near the fireplace, and in rural Bolivia, the prevalence rate was 23% in a nonsmoking community that cooked primarily indoors with cow dung (Albalak et al. 1999; Pandey et al. 1985). Case–control studies have demonstrated that wood smoke exposure is an independent risk factor for the development of COPD, with odds ratios in the range of 4–15 (Dennis et al. 1996; Perez-Padilla et al. 1996). In a cohort of Colombian women, the population attributable risk was 50% (Dennis et al. 1996).

In a 2002 WHO report (WHO 2002), the global burden of disease attributed to indoor air pollution from biomass combustion accounted for 2.7% of worldwide disability-adjusted life-years lost (Ezzati and Kammen 2002; WHO 2002), placing indoor smoke as the second largest environmental contributor to poor health, behind unsafe water and sanitation (WHO 2002). Indoor smoke accounts for 4–5% of global mortality, with 56% of these deaths due to childhood acute lower respiratory infections and the remainder due to COPD and lung cancer, primarily in women (Ezzati and Kammen 2002; WHO 2002). As the global burden of COPD continues to rise, projected to rank as the fifth most burdensome condition by 2020 (Pauwels et al. 2001), and poverty persists, we can assume the burden of disease due to biomass combustion will also continue to rise (Bruce et al. 2000).

Patients with hut lung can present with a wide spectrum of symptoms, ranging from quite benign to severe. This case demonstrates that a productive cough and mild dyspnea can persist for years after removal of the exposure. In the first published series of 25 rural South African women, most of the women were asymptomatic, and the remainder either had acute cough or a chronic productive cough (Grobbelaar and Bateman 1991). In contrast, in a later series of 30 rural Mexican women with pulmonary hypertension and cor pulmonale, all of the women had dyspnea and nearly all had a productive cough. Other common findings included cyanosis (63%), crackles (70%), hepatomegaly (60%), and edema (73%) (Sandoval et al. 1993). In the above series, because the mean age of the Mexican women was higher than the South African women (63 years vs. 43 years, respectively), it is likely that they had substantially greater cumulative exposures to smoke. This suggests that early disease can be masked by the lack of or nonspecific nature of symptoms and that duration of exposure is correlated with the severity of disease.

Pulmonary function tests also demonstrate a wide spectrum of abnormalities. The present case demonstrates that airflow obstruction can be one of the initial physiologic changes. In the previous case series, most South African women had mild and moderate airway obstruction (16 of 22) and a decreased diffusion capacity (13 of 17), whereas the remainder had normal (5 of 22) or mild restriction (1 of 22) (Grobbelaar and Bateman 1991). Although most of the Mexican women also had obstruction (23 of 30), many also had mild restriction or a mixed picture (18 of 30). Arterial blood gases demonstrated severe hypoxemia in all patients and some with hypercapnea (12 of 30) (Sandoval et al. 1993). Diffusing capacity was not measured. This suggests that early disease can be masked by normal pulmonary function tests, that airflow obstruction and impaired diffusion capacity are the initial physiologic changes, and that at late stages mild restriction and gas exchange abnormalities develop. Deterioration of pulmonary function also seems to be correlated with the duration and intensity of exposure.

Characteristic findings have been reported on bronchoscopy and bronchoalveolar lavage. In this case, gross inspection of the large airways showed dark blue stains that were similar to the airway findings described in the Mexican series (Sandoval et al. 1993). Our case also revealed normal lavage fluid cell differential counts and carbon-laden alveolar macrophages, as was reported in the South African series (Grobbelaar and Bateman 1991).

The characteristic but nonspecific plain chest radiographic findings of hut lung are diffuse pulmonary nodules. Our case illustrates that on HRCT these nodules are distributed along the bronchovascular bundles and can coexist with mediastinal lymphadenopathy. In a study from Turkey (Kara et al. (2003), a comparison of HRCT scans of 60 nonsmoking women with at least 10 years of biomass exposure with nonexposed controls showed significantly more of the following abnormalities: reticulation, peribronchovascular thickening, and nodular and ground glass opacities. The asymptomatic subjects with exposure had significantly more ground-glass opacities and less bronchiectasis than those with symptoms (Kara et al. 2003). These data suggest that radiographic abnormalities are seen early in the disease, even in asymptomatic or mildly symptomatic individuals, and persist years after removal from exposure.

Lung histopathology obtained by either transbronchial or open lung biopsy is the gold standard for the diagnosis of hut lung. This case illustrates the classic findings of carbon pigment deposition around terminal bronchioles, dust macules, and mixed dust fibrosis. In the South African series, Grobbelaar and Bateman (1991) described three patterns: isolated carbon deposition (12 of 25), macules as carbon pigment within focal collections of dust laden macrophages (6 of 25), and mixed dust fibrosis as stellate interstitial fibrous lesions (7 of 25). In the Mexican series, Sandoval et al. (1993) observed mixed dust fibrosis on lung biopsies, whereas they observed CB on airway biopsies. The available data again suggest that histopathologic changes are seen early in disease, even in asymptomatic or mildly symptomatic individuals, and persist years after removal from exposure.

## Conclusion

Hut lung appears to represent the non-infectious, nonmalignant respiratory manifestations of chronic, high level exposures to biomass smoke. There is strong evidence that chronic exposure to high levels of smoke from the combustion of biomass indoors is a risk factor for the development of CB and COPD in women of the developing world and growing evidence that an interstitial lung disease characterized by carbon deposition, dust macules, and mixed dust fibrosis also exists. This case is the first report of hut lung associated with charcoal production. The literature suggests that hut lung is likely to be part of a spectrum of disease in which intensity and duration of exposure affects the disease manifestation. Patients with early disease are either asymptomatic or present with cough or mild COPD, but radiographically and pathologically may have significant abnormalities, including fibrosis. Research is needed to better characterize disease mechanism, progression, and interventions for prevention and treatment. As residents of lesser developed countries continue to be exposed to high levels of biomass smoke at home or at work and continue to immigrate to developed countries, it is important that health care providers in developed countries learn to recognize this clinical entity.
